# Cross-Species Meta-Analysis of Transcriptomic Data in Combination With Supervised Machine Learning Models Identifies the Common Gene Signature of Lactation Process

**DOI:** 10.3389/fgene.2018.00235

**Published:** 2018-07-12

**Authors:** Mohammad Farhadian, Seyed A. Rafat, Karim Hasanpur, Mansour Ebrahimi, Esmaeil Ebrahimie

**Affiliations:** ^1^Department of Animal Science, Faculty of Agriculture, University of Tabriz, Tabriz, Iran; ^2^Department of Biology, University of Qom, Qom, Iran; ^3^Adelaide Medical School, Faculty of Health and Medical Sciences, The University of Adelaide, Adelaide, SA, Australia; ^4^Institute of Biotechnology, Shiraz University, Shiraz, Iran; ^5^Division of Information Technology, Engineering and the Environment, School of Information Technology & Mathematical Sciences, University of South Australia, Adelaide, SA, Australia; ^6^School of Biological Sciences, Faculty of Science and Engineering, Flinders University, Adelaide, SA, Australia

**Keywords:** milk production, meta-analysis, microarray, gene ontology, gene network, data mining

## Abstract

Lactation, a physiologically complex process, takes place in mammary gland after parturition. The expression profile of the effective genes in lactation has not comprehensively been elucidated. Herein, meta-analysis, using publicly available microarray data, was conducted identify the differentially expressed genes (DEGs) between pre- and post-peak milk production. Three microarray datasets of Rat, Bos Taurus, and Tammar wallaby were used. Samples related to pre-peak (*n* = 85) and post-peak (*n* = 24) milk production were selected. Meta-analysis revealed 31 DEGs across the studied species. Interestingly, 10 genes, including *MRPS18B*, *SF1*, *UQCRC1*, *NUCB1*, *RNF126*, *ADSL*, *TNNC1*, *FIS1*, *HES5* and *THTPA*, were not detected in original studies that highlights meta-analysis power in biosignature discovery. Common target and regulator analysis highlighted the high connectivity of *CTNNB1*, *CDD4* and *LPL* as gene network hubs. As data originally came from three different species, to check the effects of heterogeneous data sources on DEGs, 10 attribute weighting (machine learning) algorithms were applied. Attribute weighting results showed that the type of organism had no or little effect on the selected gene list. Systems biology analysis suggested that these DEGs affect the milk production by improving the immune system performance and mammary cell growth. This is the first study employing both meta-analysis and machine learning approaches for comparative analysis of gene expression pattern of mammary glands in two important time points of lactation process. The finding may pave the way to use of publically available to elucidate the underlying molecular mechanisms of physiologically complex traits such as lactation in mammals.

## Introduction

Milk is the crucial natural source of nutrients for the growth of newborn mammals. Mammary glands undergo regular but complex cell proliferation and involution cycles after maturity (Gao et al., 2013). Lactation can be classified into three main steps: (1) early lactation where milk is produced in increasing trends, (2) peak production where energy balance is negative, and (3) late lactation where persistency of lactation is important, especially in dairy animals. Early lactation has great differences in gene expression profile with the ones form the late lactation (Strucken et al., 2015). So, elucidating the genes influencing each lactation time point can assist the animal breeders to accelerate the genetic improvement of dairy animals in breeding programs. Gene expression profiling of milk at different stages of lactation may reflect the molecular events of mammary glands (Farhadian et al., 2018). To provide a better understanding of milk production, unraveling molecular events in mammary glands is necessary.

One of the most studied animals for milk trait is Wallaby (*Macropus eugenii*). Wallaby has a short pregnancy that lasts for only 26 days followed by an extended lactation period of about 300 days with a lactation peak of 200 days postpartum (Lefèvre et al., 2010). Rat is another employed animal for milk research that produces multiple litters of milk during multiple gestations in a short period of time. In rat, peak lactation is around 12th day postpartum (Delongeas et al., 1997; Hadsell et al., 2012). In the context of animal breeding, peak lactation of dairy cow occurs 60–90 days postpartum. The gene expression data from wallaby, rat and cow can provide useful information for accurate discovery of key genes that control milk production. In line with this argument, the study of gene expression in mouse has facilitated the identification of candidate genes of milk production in cattle (Ron et al., 2007).

Important biological processes are often precisely conserved across related species (McCarroll et al., 2004; Wang and Rekaya, 2009). Meta-analysis and machine learning have the potential to uncover the common biosignature among mammals (Shekoofa et al., 2014; Ebrahimie et al., 2018; Farhadian et al., 2018; Sharifi et al., 2018). Recently, with availability of cross-species data, meta-analysis has been performed on multiple species (Lu et al., 2009). Individual studies have some limitations in their statistical power and reliability of the results. Meta-analysis, by combining data and results of different research, improves the statistical power and accuracy of expression estimates (Ramasamy et al., 2008; Sharifi et al., 2018). Transcriptomic meta-analysis can be classified into two types: co-expression meta-analysis and expression meta-analysis. Co-expression meta-analysis investigates whether genes co-expressed in one species are also co-expressed in another species. In contrast, expression meta-analysis investigates the commonality between expression profiles of homologous genes in different species. Significant strength of co-expression meta-analysis is that microarray experiments of different species can be combined even under different experimental conditions (Lu et al., 2009).

Attribute weighting (feature selection) models, artificial neural network, deep learning, and decision trees are the main algorithms for knowledge discovery and prediction (Ebrahimi and Ebrahimie, 2010; Ashrafi et al., 2011; Ebrahimi et al., 2011; Shekoofa et al., 2011). Data mining methods are still expected to bring more fruitful results (Matsumoto, 1998; Hsiao et al., 2006; Shekoofa et al., 2011).

The aim of this study was to use meta-analysis and machine learning approaches together to increase the power of detecting the conserved genes in milk production across three different species of Wallaby, Rat, and Cow. We examined gene expression pattern of mammary gland in early and late lactation of mentioned species. Then, downstream analyses including gene ontology and gene network were performed for better understanding of the identified signature.

## Materials and Methods

### Dataset Collection and Data Preprocessing

Gene Expression Omnibus (GEO) database^1^ was used as a source of transcriptomic data collection. Datasets with biological samples for both pre- and post-peak milk production as well as their corresponding raw gene expression and annotation data were collected for meta-analysis. The general information regarding the obtained datasets is presented in **Table 1**. The datasets belonged to three different species including Wallaby, Rat, and Cow.

**Table 1:** The original datasets selected for meta-analysis of milk production.

GEO ID	No. of samples		Platform	Reference	RNA source
	Pre-peak	Post-peak			
GSE44112	3	3	Agilent-014879 Whole Rat Genome Microarray 4x44K G4131F (Feature Number version)	Izumi et al., 2014	Milk whey
GSE19055	16	15	UIUC Bos taurus 13.2K 70-mer oligoarray (condensed)	Bionaz et al., 2012	Mammary gland
GSE63654	66	6	Tammar wallaby custom 13,440 spot cDNA array	Vander Jagt et al., 2015	Mammary gland

*GEO, gene expression omnibus; GES, gene expression series; No, number.*

The first dataset (GSE44112) had 10 biological samples from three rats in three stages of lactation (on days 2, 9, and 16 postpartum) as well as one sample from serum. Samples belonging to the second day and the serum were excluded from the analyses. This dataset was one-color microarray data from rat milk whey. The microarray slides were scanned by Agilent DNA Microarray Scanner (Agilent Technologies) and Quantile method was applied to normalize the data.

The second dataset (GSE19055) contained 60 mammary biopsy samples in nine different time points from multiparous Holstein dairy cattle breed (*n* = 8). The samples were collected at 30 (*n* = 7) and 15 (*n* = 8) days before parturition, at days of 1 (*n* = 8), 15 (*n* = 8), 30 (*n* = 8), 60 (*n* = 6), 120 (*n* = 6), 240 (*n* = 5) and 300 (*n* = 4) of lactation. Samples belonging to 30 and 15 days before parturition and samples of 1 and 60 days after parturition were excluded from the analysis. Microarray type of this dataset was two-color. Background subtraction for background correction, Loess for within array normalization and Quintile for between array normalization methods were applied on the data.

The third dataset (GSE63654) had 96 mammary gland samples in four separate points of early and late pregnancies, before peak (at days of 62, 87, 110, 130, 151, 171, and 193) and late lactation (at days of 216, 243, and 266 of lactation) from wallaby. The samples of early and late pregnancy were excluded from the analyses. This dataset was a two-color microarray. Normexp + offset (for background correction), Loess (for within array normalization) and Quantile (for between array normalization) methods were applied for normalization.

The identified outlier samples were excluded from further analysis. Clustering of the samples was also carried out to ensure a clear stratification of them into the two specified stages of the lactation (pre- and post-peak milk production). R package of Limma was employed for preprocessing of data including background correction, between and within normalization, and final probe summarization (Gautier et al., 2004; Ritchie et al., 2015). Then, probe-to-gene mapping was carried out to convert probe-set expression levels into gene expression levels according to the corresponding chip datasets (Irizarry et al., 2003).

### Gene Matching

Probe IDs from different platforms were matched with their corresponding official gene symbols. Among these probe IDs, the ID with the largest interquartile range (IQR) of expression value was selected to represent the gene symbol when multiple probe IDs were matched to the same gene symbol. The IQR-based method is more robust and biologically more acceptable than the mean-based method (Hahne et al., 2010). Furthermore, in the cases that multiple probes matched a single gene, IQR-based method was used for selecting the probe (Wang et al., 2012).

### Gene Merging

**FIGURE 1:**
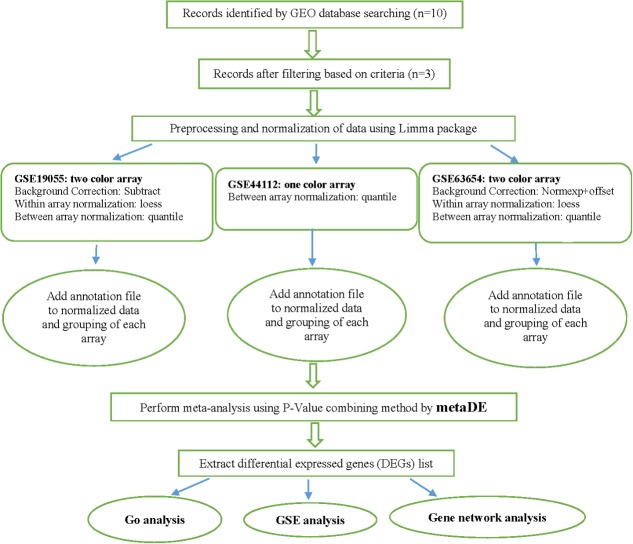
Flowchart of the performed meta-analysis of milk production in this study.

Since the number of genes in the studies were different, the multiple gene expression datasets may not be aligned by genes correctly. So, common genes across multiple studies gathered together to make the merged datasets. When a large number of studies are combined, the number of common genes may be very small. To deal with this shortcoming, we allowed a gone to be present in the analysis when is present in at least 66.66% of the studies. The steps of data preparation and meta-analysis are shown in **Figure 1**.

### Meta-Analysis

Meta-analysis can be performed based on “combining effect size,” “combining ranks” or “combining *P*-value” (Sharifi et al., 2018). Each of meta-analysis methods has different approaches for different purposes. The employed approach in this study was to analyze each experiment separately and then perform meta-analysis based on the obtained *p*-values in the individual experiments. For gene merging, we used the threshold that a gene has to be present in at least 2 out of 3 (66.66%) of experiments. The normalized datasets were used for meta-analysis. The datasets were merged using the “metaDE” package (Li and Tseng, 2011). The “combining P-value” was selected for meta-analysis of the current work. This technique sums the logarithm of the (one-sided hypothesis testing) *p*-values across k studies for a given gene. The statistic test of chi-square distribution was used with 2 degrees of freedom.

**FIGURE 2:**
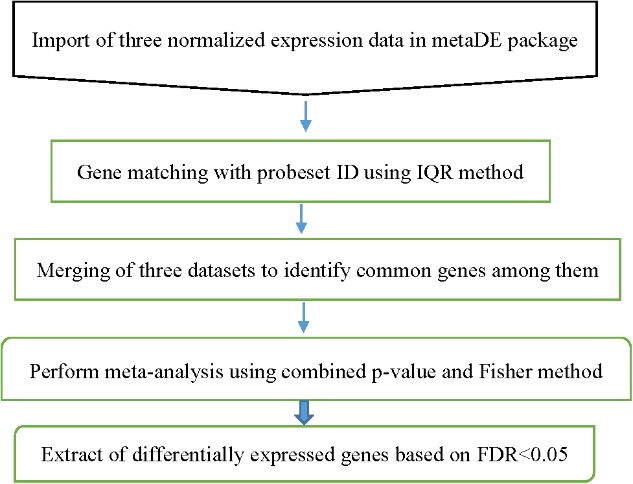
Flowchart of the different steps of milk microarray meta-analysis based on combining *P*-value strategy.

Before performing the meta-analysis, a set of *p*-values for each dataset was estimated. The metaDE package provides functions for conducting 12 major meta-analysis methods for differential expression analysis. To obtain a set of *p*-value estimates in the original individual analysis, the moderated-t statistics was used. In order to determine up- and down regulated gene after meta-analysis, the one-tailed *p*-value analysis was used in individual studies. The Fisher’s method was used for performing meta-analysis. We used permutation method (*n* = 2000) for calculation of the *p*-values. We used false discovery rate (FDR) corrected *p*-values (*P* < 0.05) to determine DEGs between the two specified stages of lactation (Benjamini and Hochberg, 1995). The flowchart of meta-analysis is shown in **Figure 2**.

### Gene Ontology (GO) Analysis

Gene ontology analysis was performed on the DEGs provided by meta-analysis based on Molecular function (MF), biological process (BP), and cellular component (CC) terms. For interpretation of the data, the GO profile of a subset of genes was compared to the GO profile of the reference set. Whole genome annotation was considered as background and FDR of 0.05 was considered as cut-off threshold of statistical significance. The String and comparative GO web tools were used to perform this task (Fruzangohar et al., 2013, 2017; Szklarczyk et al., 2014; Ebrahimie et al., 2017).

### Network Analysis

The genes/proteins functions and their underlying pathways play the key role in better understanding of the dynamic process of complex traits such as milk production in mammals. Pathway Studio was used for constructing the networks, as previously described (Hosseinpour et al., 2012; Ebrahimie et al., 2015; Pashaiasl et al., 2016; Pashaei-Asl et al., 2017). Pathway Studio has a powerful database of mammalian gene/protein/small-RNA interactions, collected by literature mining (Nikitin et al., 2003).

The network for DEGs was constructed using two algorithms of common regulation and target (Alanazi and Ebrahimie, 2016). Downstream targets that are regulated by at least two or more of the selected entities in the network diagram are found by common target algorithm. In the other ways, upstream regulators that regulate two or more of the selected entities in the network can be discovered by common regulation algorithm. Two types of entities including small molecules and proteins along with some different types of relations such as expression, promoter binding, regulation and etc. were selected to provide a comprehensive view on milk production pathways. In final network, we kept only those relations that the number of references were more than 15 for both algorithms. The Excel format of each network, including all relations and entities of the networks are recorded and presented as Supplementary Files.

### Data Mining (Supervised Machine Learning Models)

The issue of data heterogeneity from various sources (called batch effect) and their effects on meta-analysis outcome is the main concern in meta-analysis and needs to be addressed. In this study, we used 10 attribute weighting algorithms, as supervised machine learning models, to investigate the repeatability of discriminating genes between pre- and post-peak milk production in three species (Wallaby, Rat, and Cow). To test whether the developed meta-gene signature of lactation is not species independent, we used two approaches.

At first approach, attribute weighting models were run for each species separately, while pre- and post-peak milk production status was set as the target (label) variable. Then, the commonality (intersection) of discriminating in three species were identified as species-independent signature of lactation process.

In the second approach, at first, expression data of genes were standardized. Then, the expression values as well as type of species (Wallaby, Rat, and Cow) were set as the variable (feature) for attribute weighting models while the pre- and post-peak milk production status was set as the target (label) variable. In other words, this analysis will identify the most informative genes features contributing to the type of organism. The result of this analysis can address whether the developed gene signature is species-independent or species-dependent. On other words, this analysis finds whether species announces as important discriminating feature of lactation process or not.

Different algorithms of attribute weighting (feature selection) models (Information gain, Information gain ratio, Chi Squared, Deviation, Rule, SVM, Gini index, Uncertainty, Relief and PCA) were applied for the above mentioned approaches. For attribute weighting, datasets of these genes were imported into Rapid Miner software (Rapid Miner 5.0.001, Dortmund, Germany), as previously described (Ebrahimi et al., 2011, 2015; Shekoofa et al., 2011; Jamali et al., 2016). The main idea of attribute weighting was to select a subset of input features (variables) by eliminating features with little or no distinguishing information. Application of attribute weighting enables more complex data to be analyzed. Attribute weighting, as a supervised learning model finds a good for discrimination of levels of target variable. The importance value of each feature calculates as (1- p) where *p* was the *p* value of the appropriate test (Information gain, Information gain ratio, Chi Squared, Deviation, Rule, SVM, Gini index, Uncertainty, Relief, and PCA) between the candidate predictor and the lactation status.

## Results

### Meta-Analysis

After searching the microarray data repositories, we selected three expression datasets with 85 biological samples related to pre-peak and 24 biological samples related to post-peak stages of lactation.

The probe IDs from different platforms required to be matched with unique gene IDs. Thus, gene symbols were chosen to match the probe IDs. This step reduced the dimension of input matrices to a half. Finally, a total of 2,519 common genes remained among the three datasets (**Supplementary Data Sheet S1**) to be analyzed. Using Fisher method, a total of 31 DEGs (24 up-regulated and 7 down-regulated) were discovered different between the pre- and post-peak milk production. As compared to the post-peak, the top up-regulated gene in pre-peak was *ATP5B* (*P* = 0.009), while the top down-regulated gene was *CTNNB1* (*P* = 0.01).

Ten, out of 31 DEGs, were identified only by the current meta-analysis and not in the original studies. These include four down-regulated (*TNNC1*, *FIS1*, *HES5* and *THTPA*) and six up-regulated (*MRPS18B*, *SF1*, *UQCRC1*, *NUCB1*, *RNF126* and *ADSL*) genes. The detailed information of the discovered DEGs is reported in **Table 2**.

**Table 2:** The detailed information of the discovered differentially expressed genes via meta-analysis in lactation process.

Gene ID	Gene symbol	Official full name	Class	Cellular location	*P*-value
**Up-regulated genes**					
327675	*ATP5B*	ATP synthase, H+ transporting, mitochondrial F1 complex, beta polypeptide	Protein	Mitochondrion	0.009
281173	*FTH1*	Ferritin heavy chain 1	Protein	Cytoplasm	0.011
616317	*STMN1*	Stathmin 1	Protein	Cytoplasm, cytoskeleton	0.008
445425	*TKT*	Transketolase	Protein	Cytoplasm	0.014
507924	*LAS1L*	LAS1 like, ribosome biogenesis factor	Transcription Factor	Nucleus	0.021
281418	*PPIA*	Peptidylprolyl isomerase A	Receptor	Cytoplasm	0.021
531676	*KDELR2*	KDEL endoplasmic reticulum protein retention receptor 2	Receptor	Endoplasmic reticulum membrane	0.018
286853	*RPLP2*	Ribosomal protein lateral stalk subunit P2	Protein	Cytoplasm	0.033
510824	*MRPS18B*	Mitochondrial ribosomal protein S18B	Protein	Mitochondrion	0.048
281831	*HSPA8*	Heat shock protein family A (Hsp70) member 8	Protein	Cytoplasm	0.018
541229	*SF1*	Splicing factor 1	Protein	Nucleus	0.045
507309	*VAMP8*	Vesicle associated membrane protein 8	Receptor	Lysosome membrane	0.018
617534	*RSU1*	Ras suppressor protein 1	Protein	Cytoplasm	0.046
535273	*EMP3*	Epithelial membrane protein 3	Protein	Membrane	0.013
282379	*TAGLN2*	Transgelin 2	Protein	Cytoplasm	0.019
282393	*UQCRC1*	Ubiquinol-cytochrome c reductase core protein I	Protein	Mitochondrion inner membrane	0.033
507672	*FOLR2*	Folate receptor 2 (fetal)	Receptor	Cell membrane	0.033
505351	*NUCB1*	Nucleobindin 1	Protein	Golgi network membrane	0.039
281997	*PRDX1*	Peroxiredoxin 1	Protein	Cytoplasm	0.048
507447	*RNF126*	Ring finger protein 126	Protein	Cytoplasm	0.028
282290	*NDUFV2*	NADH:ubiquinone oxidoreductase core subunit V2	Protein	Mitochondrion inner membrane	0.022
510949	*ADSL*	Adenylosuccinate lyase	Protein	Cytoplasm	0.043
280994	*ALPL*	Alkaline phosphatase, liver/bone/kidney	Protein	Cell membrane	0.027
505997	*SPNS1*	Sphingolipid transporter 1	Transporter	Mitochondrion inner membrane	0.035
**Down-regulated genes**					
539003	*CTNNB1*	Catenin beta 1	Transcription Factor	Cytoplasm	0.01
615565	*FIS1*	Fission, mitochondrial 1	Protein	Mitochondrion outer membrane	0.014
509486	*TNNC1*	Troponin C1, slow skeletal and cardiac type	Protein	Cytoplasm	0.018
281057	*CD44*	CD44 molecule (Indian blood group)	Receptor	Cell membrane	0.018
787633	*HES5*	Hes family bHLH transcription factor 5	Transcription Factor	Nucleus	0.094
280843	*LPL*	Lipoprotein lipase	Protein	Cell membrane	0.021
282090	*THTPA*	Thiamine triphosphatase	Protein	Cytoplasm	0.036

### Functional Annotation and Pathway Analysis

Gene ontology enrichment analysis was performed to achieve the better understanding of the biological roles of the DEGs on lactation process. There were 55 significant enriched GO terms (31, 4 and 20 for CC, MF and BP categories, respectively). The two top significantly enriched BPs were single-organism cellular process (GO: 0044763, *P* = 0.000192) and single-organism process (GO: 0044699, *P* = 0.000944). In CC category, the two top enriched terms were vesicle (GO: 0031982, *P* = 3.47E-05) and extracellular exosome (GO: 0070062, *P* = 3.47E-05). The two most significantly enriched MFs were binding and ion binding. The significantly enriched GO terms of the DEGs are reported in **Table 3**.

**Table 3:** The enriched Gene Ontology (GO) terms of differentially expressed genes discovered via meta-analysis between pre- and post-peak milk production.

GO ID	GO Names	GO Terms	FDR
GO.0044763	Single-organism cellular process	BP	0.000192
GO.0044699	Single-organism process	BP	0.000944
GO.0032879	Regulation of localization	BP	0.00133
GO.0065008	Regulation of biological quality	BP	0.00133
GO.0044710	Single-organism metabolic process	BP	0.00238
GO.0042592	Homeostatic process	BP	0.00952
GO.0032880	Regulation of protein localization	BP	0.014
GO.0051049	Regulation of transport	BP	0.0167
GO.0008152	Metabolic process	BP	0.0186
GO.0050789	Regulation of biological process	BP	0.0186
GO.1903827	Regulation of cellular protein localization	BP	0.0186
GO.0009987	Cellular process	BP	0.0262
GO.0019637	Organophosphate metabolic process	BP	0.0274
GO.0032386	Regulation of intracellular transport	BP	0.0274
GO.0060341	Regulation of cellular localization	BP	0.0274
GO.2000179	Positive regulation of neural precursor cell proliferation	BP	0.0274
GO.0044237	Cellular metabolic process	BP	0.0275
GO.0016192	Vesicle-mediated transport	BP	0.0304
GO.0006810	Transport	BP	0.0386
GO.0022411	Cellular component disassembly	BP	0.0386
GO.0005488	Binding	MF	8.54E-06
GO.0043167	Ion binding	MF	0.00257
GO.0003824	Catalytic activity	MF	0.00378
GO.0046872	Metal ion binding	MF	0.011
GO.0031982	Vesicle	CC	3.47E-05
GO.0070062	Extracellular exosome	CC	3.47E-05
GO.0043226	Organelle	CC	0.000192
GO.0005623	Cell	CC	0.000228
GO.0044464	Cell part	CC	0.000228
GO.0043209	Myelin sheath	CC	0.000685
GO.0044444	Cytoplasmic part	CC	0.000739
GO.0005739	Mitochondrion	CC	0.00095
GO.0005576	Extracellular region	CC	0.000965
GO.0016020	Membrane	CC	0.000995
GO.0031966	Mitochondrial membrane	CC	0.00102
GO.0043227	Membrane-bounded organelle	CC	0.00102
GO.0005740	Mitochondrial envelope	CC	0.00112
GO.0044425	Membrane part	CC	0.00122
GO.0071944	Cell periphery	CC	0.00292
GO.0005743	Mitochondrial inner membrane	CC	0.00332
GO.0043229	Intracellular organelle	CC	0.0038
GO.0005737	Cytoplasm	CC	0.00404
GO.0005829	Cytosol	CC	0.00414
GO.0032991	Macromolecular complex	CC	0.0127
GO.0005886	Plasma membrane	CC	0.0131
GO.0005622	Intracellular	CC	0.0144
GO.0044455	Mitochondrial membrane part	CC	0.0144
GO.0044429	Mitochondrial part	CC	0.0189
GO.0043231	Intracellular membrane-bounded organelle	CC	0.0242
GO.0005925	Focal adhesion	CC	0.0257
GO.0031090	Organelle membrane	CC	0.0257
GO.0031224	Intrinsic component of membrane	CC	0.0267
GO.0043232	Intracellular non-membrane-bounded organelle	CC	0.0316
GO.0031225	Anchored component of membrane	CC	0.0319
GO.0044424	Intracellular part	CC	0.0324

*Only the significantly enriched (*P* ≤ 0.05) GO terms are presented. BP, biological process; CC, cellular component; MF, molecular function.*

### Network Analysis

#### Sub-network Discovery in DEGs

Genes do not act solely but interact with other cell elements in order to make the cell activities more efficient. Genes that interact with each other generate a sub-network and two or more sub-networks join each other to make a network. So, detection of significant sub-networks is an important task in network analysis. To this end, we used some relations such as expression, regulation, promoter binding, direct regulation, miRNA effect, mol synthesis and chemical reaction. Statistically significant sub-networks which were generated by upstream and downstream network analysis are presented in **Supplementary Data Sheets S2**, **S3**, respectively.

**FIGURE 3:**
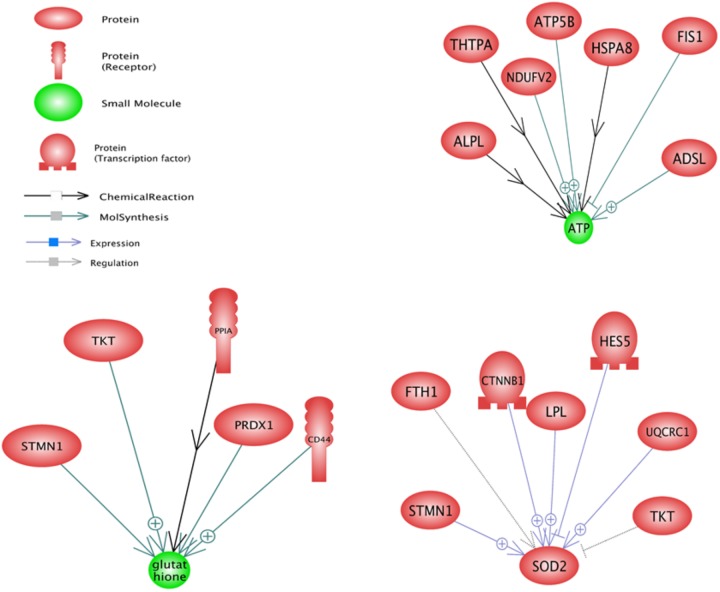
Significant upstream sub-networks constructed by differentially expressed genes between pre-peak and post-peak milk production. ⊕ Represents positive-regulated and ⊢ represents negative-regulated. Glutathione, SOD2, and ATP were the three top important sub-networks.

In upstream level, sub-networks of glutathione, SOD2, and ATP were three top important sub-networks (**Figure 3**). Glutathione and ATP sub-networks were the two most enriched small molecules that were enriched with DEGs. *TKT* and *STMN1* were the two genes that affect the glutathione and SOD2 sub-networks. SOD2 sub-network is regulated by two transcription factors named *CTNNB1* and HES5.

**FIGURE 4:**
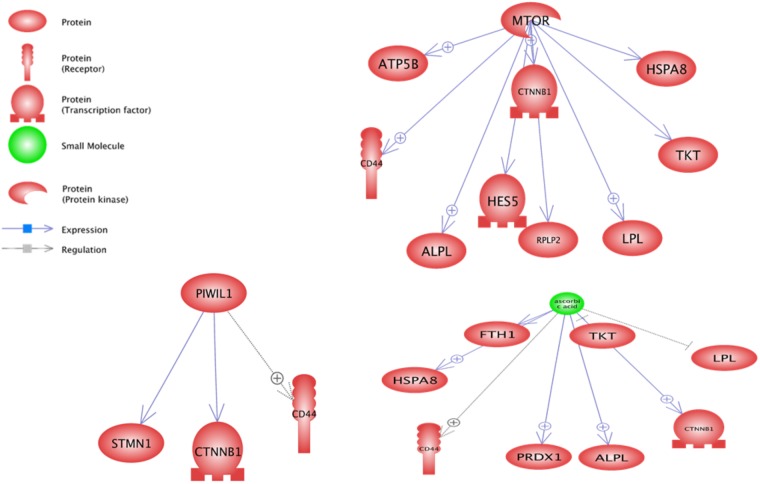
Significant downstream sub-networks constructed by differentially expressed genes between pre-peak and post-peak milk production. ⊕ Represents positive-regulated and ⊢ represents negative-regulated. PIWIL1, Ascorbic Acid and MTOR were the most important sub-networks.

In downstream level, the PIWIL1, Ascorbic Acid and MTOR were the most important sub-networks (**Figure 4**). Ascorbic Acid was the major small molecular for regulation of some genes including *TKT*, *LPL*, *CD44*, *FTH1*, *PRDX1*, *HSPA8*, *CTNNB1* and *ALPL*.

Based on the sub-network results, especially downstream analysis, the *CTNNB1* and *CD44* genes contributed in three most enriched sub-networks and were under the control of PIWIL1, Ascorbic Acid and MTOR. Also *TKT*, *ALPL*, *LPL* and *HSPA8* were under the control of Ascorbic Acid and MTOR. Probably, a gene under the control of more than one regulator plays a key function in cell. There were some other enriched downstream sub-networks such as glucose, cysteine, vitamin D, Ca2+, Fe2+, and Mg2+ along with some microRNAs including m_Mir709, MIR100, MIR590, and MIR655 that are shown in **Supplementary Data Sheet S3**.

#### Network Analysis of DEGs in Before Versus After Milk Peak Production

**FIGURE 5:**
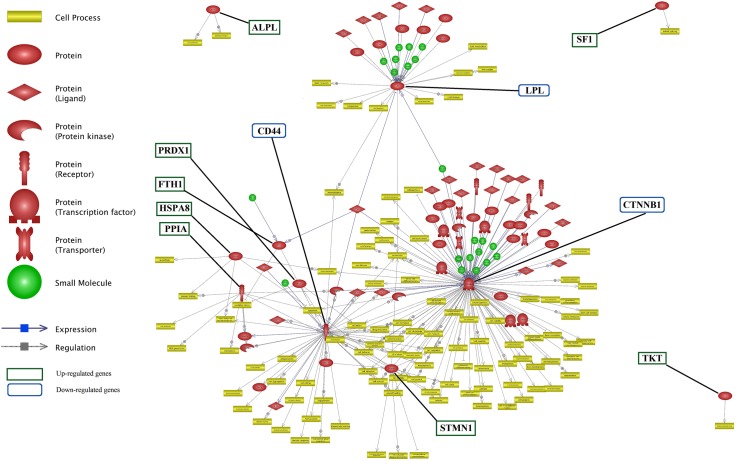
Network for differentially expressed genes involved in lactation process. The green and blue boxes are up- and down-regulated genes interactions, respectively. *CTNNB1, CD44, STMN1*, and *LPL* genes from down-regulated genes list and *TKT, SF1*, and *ALPL* from up-regulated genes list have the most number of interactions.

Network analysis was performed to construct the possible networks of the DEGs using neighbor joining algorithm (**Figure 5**). Additional information about this network is presented in **Supplementary Data Sheet S4**.

*TKT*, *SF1* and *ALPL* were up-regulated genes without any connection to the main network while each influenced a specific cell processes. Whereas, genes such as *CTNNB1*, *CD44*, *STMN1* and *LPL* were down-regulated genes with a considerable number of interactions, as compared with the remaining genes in the network.

**FIGURE 6:**
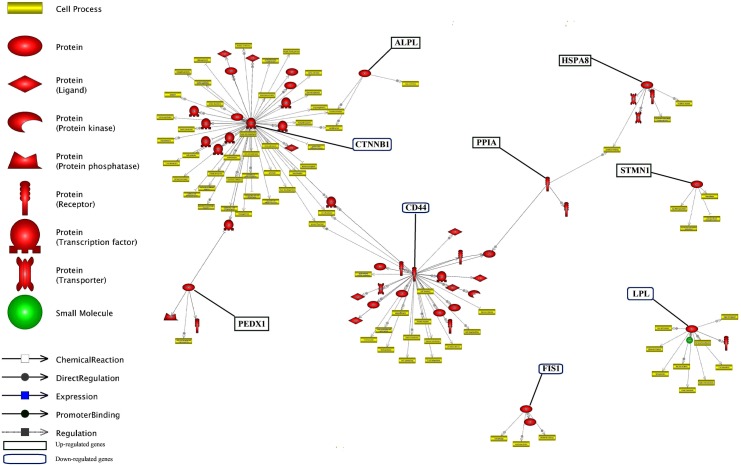
Common target analysis between differentially expressed genes in lactation process. The green and blue boxes are up- and down-regulated genes, respectively. *CTNNB1, CD44*, and *LPL* genes have the most common target.

Unraveling the common targets of the DEGs is an important issue in network analysis. Common target analysis showed that the *CTNNB1* and *CD44* genes had the highest number of common targets (**Figure 6** and **Supplementary Data Sheet S5**). Cross talk between six nodes (*CTNNB1*, *CD44*, *ALPL*, *PRDX1*, *PPIA* and *HSPA8* genes) is presented in **Figure 6**. *CTNNB1* and *CD44* connected each other via their three common targets. In addition, *CTNNB1* and *PRDX1* connected each other via one transcription factor as a common target. *LPL* did not have any target commonly with other genes but, it had the highest number of common targets among the unconnected nodes.

**FIGURE 7:**
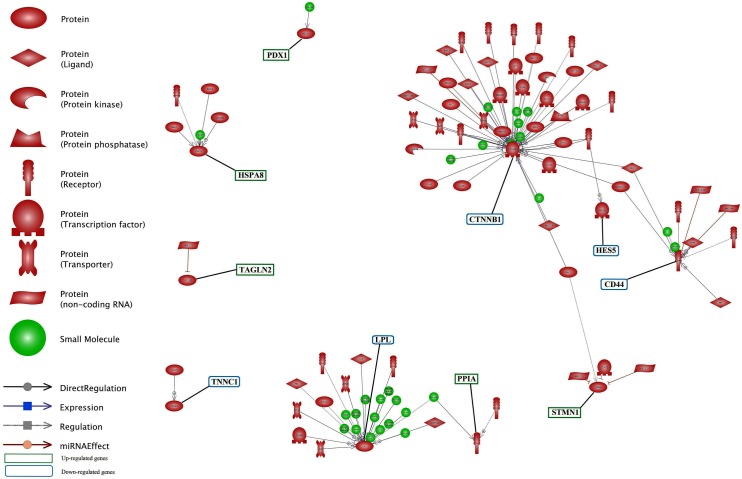
Common regulation analysis between DEGs in lactation process. The green and blue boxes are up- and down-regulated genes, respectively. *CTNNB1*, *CD44* and *LPL* genes have the most common regulation.

The identification of common regulation of genes is important in gene networking. The common regulation entities of DEGs is presented in **Figure 7** and **Supplementary Data Sheet S6**. Down-regulated genes of *CTNNB1*, *CD44* and *LPL* along with up-regulated genes of *HSBA8*, *STMN1* had more common regulator entities. In this network, we infer the important genes, i.e., genes with more regulators. So, it can be understood that these genes play an important function in milk production, especially at later stage of lactation. Each of *TNNC1*, *TAGLN2* and *PRDX1* had only one regulator. In contrast, *LPL* had the highest number of small molecules as regulator.

#### Sub-networks Generated by DEGs

**FIGURE 8:**
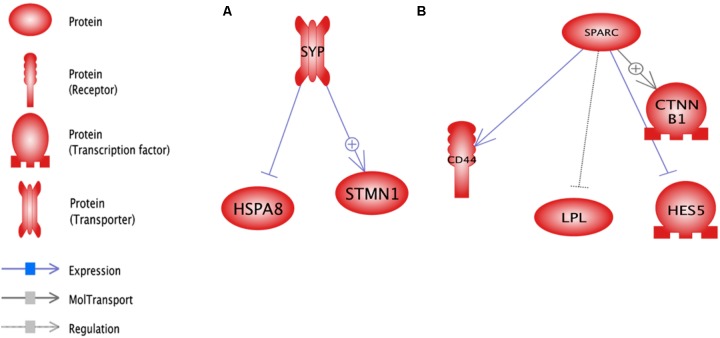
Enriched sub-networks in up-stream neighbors of differentially expressed genes in lactation process; **(A)** Down-regulated genes, **(B)** Up- regulated genes.

The analysis of significant sub-network for up and down-regulate genes was carried out using up- and down-stream categories. For each category, the significant level of 0.05 selected and maximum significant sub-network for each were 100. SPARK (*P* = 2.37E-07) and SYP (0.000125875) were the enriched sub-networks with down and up-regulated genes, respectively, (**Figure 8**). Additional information about the significant sub-networks for down and up-regulated genes are presented in **Supplementary Data Sheets S7**, **S8**, respectively.

**FIGURE 9:**
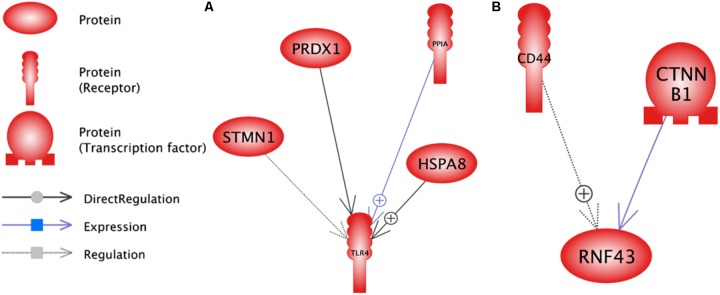
Enriched sub-networks in down-stream neighbors of differentially expressed genes in lactation process; **(A)** Down-regulated genes, **(B)** Up- regulated genes.

RNF43 (*P* = 1.4E-05) and TLR4 (*P* = 2.4E-05) were the most enriched sub-networks by down and up-regulated genes, respectively, by upstream neighbors (**Figure 9** and **Supplementary Data Sheets S9**, **S10**, respectively).

RNF43 sub-network is controlled by down-regulated genes such as *CTNNB1* as a transcription factor and *CD44* as a receptor. Furthermore, TLR4 sub-network is under the control of *HSPA8*, *PRDX1*, *STMN1* and *PPIA* genes as receptors.

The enriched sub-network for up-regulated genes using up and down-stream categories were SYP and TLR4, respectively. The *STMN1* and *HSPA8* were the common genes that involved in both sub-networks (**Figures 8**, **9**). The enriched sub-networks with up and down-stream categories using down-regulated genes (SPARK and RNF43, respectively) were similar in two genes. The *CTNNB1* and *CD44* were the down-regulated genes that exist in the sub-networks.

### Data Mining

#### Data Cleaning

Meta-analysis on datasets from three different species (Bovine, Rat, and Wallaby) determined 2519 common genes. Using some data cleaning methods such as useless attributes remover and remove correlated attributes (correlation greater than 95%), the final number of genes decreased to 215 genes.

Useless attributes were the attributes (genes) with very low variation (CV < 0.1) that could not be important in pre-peak and post-peak stage discrimination.

#### Attribute Weighting

As data was normalized before running the attribute weighting models, all resulting weights were between 0 and 1. The results of 10 different attribute weighting algorithms application on three spices (Cow, Rat, and Wallaby) are presented in **Supplementary Tables S1–S3**, respectively. Features with weights closer to 1 show the importance of each variable in regard to target label. An attribute was assumed important if the assigned weight was higher than 0.7 by a certain attribute weighting algorithm (**Supplementary Tables S1–S3**).

The number of attribute weighting algorithms that supported the selected DEGs are presented in **Table 4**. The complete list for all common genes are shown in **Supplementary Data Sheet S10**.

**Table 4:** Machine learning models based on attribute weighting models demonstrated that the developed transcriptomic signature of lactation is independent from the species.

Attribute	The number of attribute weighting algorithms that indicated the DEGs algorithm weighting
RSU1	5
MRPS18B	3
PPIA	3
TAGLN2	3
ATP5B	3
VAMP8	3
THTPA	3
FTH1	3
RPLP2	3
LAS1L	3
RNF126	3
EMP3	3
STMN1	3
KDELR2	3
HSPA8	3

*Here, type of species (Tammar Wallaby, Rat, and Cow) was included in analysis as well as expression levels of genes. The number of attribute weighting for differentially expressed genes and organism by different attributes weighting algorithms higher than 0.5. Total number of attribute weighting algorithms which have announced the certain attribute important (weight higher than 0.5, **Supplementary Data Sheet S11**). This table presents the number of algorithms that selected the attribute. Weighting algorithms were Uncertainty, Gini index, Chi Squared, Rule, Information Gain, and Information Gain Ratio.*

From 76 DEGs in cow dataset (GSE19055), 18 of them were also selected as DEGs by meta-analysis DEGs list; while the numbers of DEGs from meta-analysis for rat (GSE44112) and wallaby (GSE63654) datasets were 5 and 20 DEGs (out of 5 and 174 genes for each dataset, respectively). The results of meta-analysis showed 31 DEGs and 11 genes were not in any of three datasets. According to the **Table 4**, the organism weight compare with DEGs is low.

**FIGURE 10:**
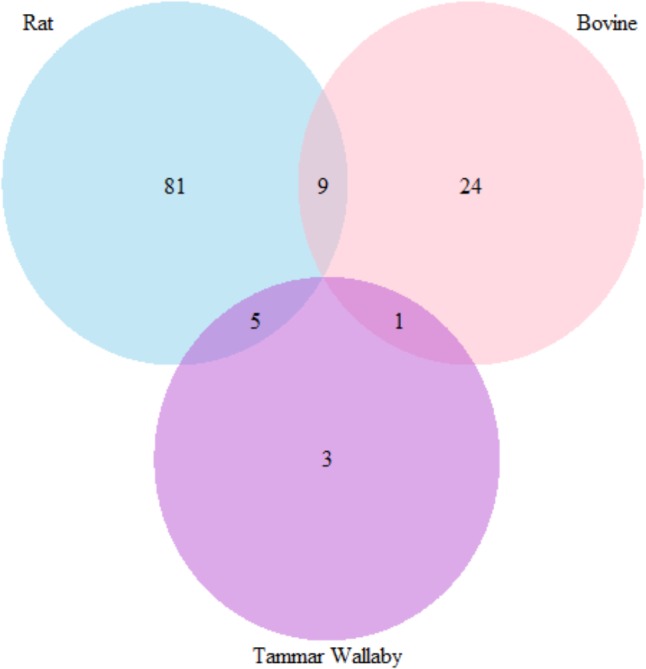
Venn diagram representing the number of genes that were selected by more than three attribute weighting model in three species to differ in lactation process.

The number of common gene which has more than three attribute weighting models with count higher than 50% in three species are presented in **Figure 10**.

The number of genes has at least three weighting models in rat, wallaby, and cow is 95, 9, and 34 respectively. There are 9 common genes between rat and bovine; 5 common genes between rat and wallaby and only 1 common gene between cow and wallaby.

## Discussion

Although vertebrates differ each other phenotypically, they share similar body plans, organs and tissues. The three selected species in this study have a range of lactation processes. Wallaby is a marsupial, with an entirely different gestation-birth-lactation system to eutherian mammals. Cow has a relatively slow single birth system and rat has a rapid birth system. However, the physiology of the mammary gland is relatively similar among mammals and there are core physiological events in the mammary gland that are similar in the mammalian species (Lu et al., 2009). Our findings show that a common transcriptome signature of lactation process exists between animals with a range of lactation system.

Nowadays, the high throughput data has enabled the researchers to discover several candidate biomarkers for various traits. Using publicly available high throughput microarray data, a meta-analysis was carried out in the current work to identify the DEGs between early (pre-peak) and late (post-peak) lactation. Meta-analysis is a powerful method for detection of the genes with small, but consistent effect on the trait of interest (Rest et al., 2016). The small-effect genes may neither be discoverable in a sole experiment nor be consistent in effect in multiple individually studied experiments. However, gathering information from multiple studies, as performed in meta-analysis, helps to discover these kind of effective genes more accurately. To our knowledge, this is the first study in which the multiple publicly available microarray datasets belonging to the two important time points of lactation were analyzed. As the main result, we identified 31 (24 up- and 7 down-regulated) DEGs between the two specified stages of lactation from which ten DEGs were novel. These novel genes include six up-regulated (*MRPS18B*, *SF1*, *UQCRC1*, *NUCB1*, *RNF126* and *ADSL)* and four down-regulated *(TNNC1*, *FIS1*, *HES5* and *THTPA)* and are reported as milk production-related DEGs for the first time in the current work.

The up-regulated gene with the lowest *P*-value was *ATP5B*. This gene has been used as a housekeeping gene in the gene expression analysis of mammary gland samples, as its expression is relatively stable across estrus cycle phases (Hvid et al., 2011). Housekeeping genes tend to keep their expression relatively constant across various tissues or conditions. However, although there is no previous report about the possible effect of this gene on milk production, the significantly over expression of *ATP5B* at early stage of lactation, as compared to later stage of lactation, suggests an important role for *ATP5B* to contribute to the differences in milk production. In line with the previous reports, we found some DEGs with direct or indirect association with milk production including *FTH1*, *TAGLN2*, *STMN1*, *TKT*, *RSU1*, *RPLP2*, *NDUFV2*, *LAS1L*, *KDELR2*, *TKT*, *PPIA*, *HSPA8*, *VAMP8*, *FOLR2*, *PRDX1* and *ALPL*. One of the most important genes express in secretary tissues, such as mammary gland, is *VAMP8* (Ren et al., 2007). The expression of *VAMP8* in the current study was significantly higher in pre-peak than the post-peak, probably due to the more milk production of secretary cells of mammary gland at earlier stage of lactation.

The lowest *P*-value among the down-regulated genes was *CTNNB1*. Wnt signaling pathway, involved in mammary growth and differentiation in mice (Shimizu et al., 1997; Howe et al., 2003; Mankertz et al., 2004; Teulière et al., 2005), is the most important pathway of *CTNNB1*. *CTNNB1* may contribute to the maintenance of milk production after peak or persistency of lactation. Among the genes related to lipid metabolism, only the expression of *LPL* was significant. A complex process take place in mammary gland (Bionaz and Loor, 2008) where milk fat content is higher at post-peak than the early stage of lactation. Higher fat content of milk sustains the young growth through supplying it the major source of energy (Green et al., 1983; Green, 1984; Kwek et al., 2007). The significantly lower expression of *LPL* pre-peak is in accordance with the findings of Green et al. (1983) and Kwek et al. (2007).

Candidate genes with known effects on the production of milk or its ingredients including *DGAT1* (Grisart et al., 2004), *GHR* (Blott et al., 2003), *SCD* (Kinsella, 1972) were not differentially expressed in the current work. Also, the most important milk protein genes such as *CSN2*, *CSN1S1*, *LGB*, *CSN3*, *CSN1S2* and *LALBA* did not have significant differential expression between the two stages of lactation. At least 22 genes are in close relation with citrate metabolism (Cánovas et al., 2013), and 31 genes encode endogenous proteases (Wickramasinghe et al., 2012; Suárez-Vega et al., 2015). None of them, however, is among the DEGs identified in this meta-analysis. This is not because these genes are less important, rather this probably means that the mentioned genes are equally important throughout the lactation.

Results of GO analysis confirmed the functional role of the DEGs on milk production. The biological importance of single-organism cellular process is in the development of mammary gland alveolus. Also, the biological function of the single-organism process related to epithelial cell proliferation involved in mammary gland duct elongation (Humphreys et al., 1997). Exosomes have been shown to package and present antigen to immune cells and have other immune modulators roles (Giri et al., 2010). In the vesicle membranes, not only the alveolar cells calcium pump activates but also glucose transport system in the mammary gland (McManaman and Neville, 2003).

Based on the results of sub-network analysis, the SOD2, glutathione and ATP sub-networks were the three most upstream enriched sub-networks. Glutathione is a small molecular that affects the immune system (Perricone et al., 2009). Also, SOD2 acts as a regulator of immunity (Scheurmann et al., 2014). In addition to the enriched sub-networks related to immunity, the function of *NUCB1* (Ma et al., 2014), *RNF126* (Delker et al., 2013), *FIS1* (Cheng et al., 2008), and *TNNC1* (Augustin et al., 2016) genes were all reported to be related to the improvement of immune system. It can be concluded that, the activation of immune system is one of the most important functions of the DEGs. Therefore, it seems that one of the ways the DEGs affect the milk production is the development of immunity. In fact, animals with strong immunity against some disease (e.g., resistant to mastitis) produce more milk than non-healthy animals.

Network analysis for detection of hub genes revealed that *CTNNB1* is a hub protein with higher number of interactions with others in the network. It is regulated by 11 small molecules. Cell proliferation, the most relevant cell process related to *CTNNB1*, has been frequently referred to in the literature (**Supplementary Data Sheet S4**). In the network, *CTNNB1* joined to *LPL* and *CD44*, which were both also central genes with a considerable number of connections. Interestingly, all of these three hub genes were down-regulated in the pre-peak rather than the post-peak. The important role of these three hub genes on the milk production was confirmed by all of the three algorithms (neighbor joining, common target, common regulation) used to construct the networks. The RNF43 had negative regulation effect on Wnt signaling pathway (Strikoudis et al., 2014). In addition, RNF43 was regulated by *CTNNB1* and *CD44*. Therefore, it can be concluded that these genes regulate Wnt signaling pathway through negative effect on RNF43 and decline the production of milk at later days of lactation. There were other DEGs that related to cell proliferation and differentiation including *SF1* (Tanaka and Nishinakamura, 2014); *UQCRC1* (Zucchi et al., 2002); *HES5* (Fathi et al., 2011); *THTPA* (Fischer-Fodor et al., 2015); *ADSL* (Skottman et al., 2005) and *MRPS18B* (Thompson-Crispi et al., 2014).

Applying 6 statistically different attribute weighting algorithms and selection of the key features based on the overall (intersection) of these algorithms reinforced the importance of the selected features. According to **Table 4**, the organism feature attribute weighting is less than the most gene features. So, we conclude that, the type of organism has lower importance in this analysis. Milk production is influenced by many factors that can be classified into genetic and non-genetic factors. Since the lactation lasts for a long time in mammalian life, there should be some genes that regulate the entire lactation by keeping their expression relatively constant throughout the lactation. While some genes may go into considerable or negligible modifications in expression during the different stages of lactation and, thus, contribute to the corresponding differences exist in milk production at different stages of lactation. We investigated the possible modifications happen in gene expression between early and late stages of lactation and found out that genes related to the development of the mammary gland, proliferation and differentiation of cells as well as genes related to the improvement of immune system were mainly altered in their expression between the specified time-points of the lactation. We conclude that the development of immunity, especially at early stages of lactation, is probably very important. Because animals are very sensitive against pathogens and diseases like mastitis at early stages of lactation. Furthermore, the activation of genes related to cell proliferation and cell differentiation sustain the growth of mammary gland, especially after peak, and help milk production to continue more persistently.

Mammals are distinguished from other animals since they produce milk for their newborn nutrition. These animals transfer some immunity-related elements to their milk in order to develop their youths‘ immune system and to protect themselves from infectious disease such as mastitis (Hasselbalch et al., 1996; Thompson et al., 2000). The developed gene signature is involved in activation of immune system and propagation of mammary gland cells as observed in other mammals (Farhadian et al., 2018).

## Conclusion

The present study was designed to identify the DEGs between two different stages (pre- and post-peak) of milk production using meta-analysis of multiple milk microarray datasets. In total, this work detected 31 DEGs in two different stage of milk production. Among DEGs, we report 10 genes for first time as candidate genes that affect milk production at different periods of lactation. Network analysis highlighted the *CTNNB1*, *CDD44* and *LPL* genes. Our study suggests that the DEGs influence on milk production by improvement of immune system and cell differentiation. Milk production is a complex trait so considerably more work will need to be performed to identify all genes related to specific time points of lactation. Using attribute weighting models and counting the species as variable in addition to gene expression levels, we showed that the developed meta-analysis signature of lactation is species-independent and is common among species. The employed approach in this study, by integrating supervised machine learning and meta-analysis, can be verified in future similar studies.

## Ethics Statement

All participants provided written and informed consent.

## Author Contributions

MF: research concept and design, data analysis and interpretation, wrote the article, and final approval of the article. SR and KH: wrote the article. ME: data analysis and interpretation and wrote the article. EE: data analysis and interpretation, critical revision of the article, and final approval of the article.

## Conflict of Interest Statement

The authors declare that the research was conducted in the absence of any commercial or financial relationships that could be construed as a potential conflict of interest.

## Footnotes

1 https://www.ncbi.nlm.nih.gov/

## Supplementary Material

TABLE S1 The results of application of 10 different attribute weighting algorithms for Bovine.

TABLE S2 The results of application of 10 different attribute weighting algorithms for rat.

TABLE S3 The results of application of 10 different attribute weighting algorithms for Wallaby.

DATA SHEET S1 Common genes among three different datasets.

DATA SHEET S2 Statistically significant subnetworks which can be generated by upstream genes.

DATA SHEET S3 Statistically significant subnetworks which can be generated by downstream genes.

DATA SHEET S4 The networks for DEGs were constructed using the neighbor joining algorithm.

DATA SHEET S5 The networks for DEGs were constructed using the common target algorithm.

DATA SHEET S6 The networks for DEGs were constructed using the common regulation algorithm.

DATA SHEET S7 Statistically significant subnetworks with downstream neighbors by down-regulated.

DATA SHEET S8 Statistically significant subnetworks with downstream neighbors by up-regulated.

DATA SHEET S9 Statistically significant subnetworks with upstream neighbors by down-regulated.

DATA SHEET S10 Statistically significant subnetworks with upstream neighbors by up-regulated.

DATA SHEET S11 The number of attribute weighting algorithm for all common genes.
