# Corrigendum: Cross-Species Meta-Analysis of Transcriptomic Data in Combination With Supervised Machine Learning Models Identifies the Common Gene Signature of Lactation Process

**DOI:** 10.3389/fgene.2019.01034

**Published:** 2019-10-16

**Authors:** Mohammad Farhadian, Seyed A. Rafat, Karim Hasanpur, Mansour Ebrahimi, Esmaeil Ebrahimie

**Affiliations:** ^1^Department of Animal Science, Faculty of Agriculture, University of Tabriz, Tabriz, Iran; ^2^Department of Biology, University of Qom, Qom, Iran; ^3^Adelaide Medical School, Faculty of Health and Medical Sciences, The University of Adelaide, Adelaide, SA, Australia; ^4^Institute of Biotechnology, Shiraz University, Shiraz, Iran; ^5^Division of Information Technology, Engineering and the Environment, School of Information Technology & Mathematical Sciences, University of South Australia, Adelaide, SA, Australia; ^6^School of Biological Sciences, Faculty of Science and Engineering, Flinders University, Adelaide, SA, Australia

**Keywords:** milk production, meta-analysis, microarray, gene ontology, gene network, data mining

In the original article, we neglected to include the funder “Iran National Science Foundation (INFS), Grant No. 95814261”.

A correction has been made to the *Funding* statement:

“The authors would like to thank the Iran National Science Foundation (INSF, Grant No. 95814261) for the financial support. We would also like to thank the authorities of Tabriz University.”

The authors apologize for this error and state that this does not change the scientific conclusions of the article in any way.

