# Supplementary material for: Cross-Species Meta-Analysis of Transcriptomic Data in Combination With Supervised Machine Learning Models Identifies the Common Gene Signature of Lactation Process
Source: Front Genet. 2018 Jul 12;9:235. doi: 10.3389/fgene.2018.00235 (PMC6052129; doi:10.3389/fgene.2018.00235)
Supplement: TABLE S1 — The results of application of 10 different attribute weighting algorithms for Bovine. [file Table_1.DOCX]

| **Model** | **Attribute** | **Weight** |
| --- | --- | --- |
| PCA | PDIA5 | 1 |
|  | IGFBP2, MRM1, SPNS1, EIF4A1 | 0.9 |
|  | AK1, C1D, BRWD1, NPC2, FTH1, FTH1, PSMC4, TNNC1, OAT | 0.8 |
|  | IMMT, LEO1, ACAT2, VAMP8 | 0.7 |
| SVM | TAGLN2 | 1.0 |
|  | HDAC3 | .9 |
| Relief | CTNNB1 | 1.0 |
|  | USP12, IFRD2 | .8 |
| Uncertainty | USP12 | 1.0 |
|  | CAPZA2, PSMC4, PDE6D | .7 |
| Gini Index | FIS1, EIF4B, | 1.0 |
|  | RPLP2 | .9 |
|  | CTNNB1, ELOF1, SEC61A1, DDX5, KDELR2, HDAC3 | .8 |
|  | ALPL, DDR1, PRDX1, USP12, GCLC | .7 |
| Chi Squared | USP12 | 1.0 |
|  | PDE6D, CAPZA2 | .8 |
|  | SCARB1 | .7 |
| Deviation | TNNC1, HGF | 1.0 |
|  | FIS1 | .9 |
|  | IGFBP2, BRWD1 | .8 |
|  | RWDD1 | .7 |
|  | PDIA5 | .7 |
| Rule | FIS1, IGFBP2, BRWD1, RWDD1, C1D, PLA2G1B, MRM1, EIF4A1, LDHA, ALAS2, LMNA, SPNS1, NPC2, ACAT2, SCARB1, STMN1, MESDC2, LEO1, GAK, OAT, IGJ, AUP1, IMMT, SLC35C2, C1QB, CCDC22, EIF5A, PSMD6, AKR1B1, CTSH, PPIA, C1QBP, DPP3, UQCRC1, USP12, PLP2, VAMP8, RPL27, CXCR4, UHRF1, COL6A2, CNOT7, CCT4, HSPB1, ACTR1A, PLD3, ATP5G3, IGFBP6, B4GALT2, LGTN, NUDT2, RPL24, ADD3, NR2F1, DDX56, GSTP1, POMT1, IFRD2, OGT, MYL2, SFMBT1, FOS, SPAG7, POLE3, SF1, NDUFV3, RPS28, AHCY, PNPLA6, PPP2CA, VPS25, SMARCB1, CLU, RPL18A, CTSZ, NR4A1, UQCRC2, PPIB, RPL10, C1S, CLDN3, CLIC1, SP1, CTSA, YY1, DNPEP, NFKBIA, ELOVL5, PLXNA2, CSNK1A1, ATP5G1, NUDCD2, CD44, PDE6D, RAP1GDS1, CCT6A, XDH, GLTSCR2, RPLP2, NAGK, NUBP2, LPL, PIM1, DAZAP2, SNW1, TEX261, ADSL, EEF1B2, OGN, POMGNT1, ADRBK1, C3, NFIA, RBMS1, ARHGAP29, METAP2, DRG1, EHF, RPS12, MTCH1, GHITM, PRDX2, SCG5, DERL1, GPAA1, FOLR2, RPS25, TKT, FLNA, G6PD, FGD1, AP3B1, PSEN2, SEC61A1, HGF, SLC39A7, LUM, PRDX5, FUS, ELOF1, DDX50, CTNNB1, ATP2C1, KDELR2, SLC25A11, CTSC, SPRY1 | 1.0 |
| Info Gain Ratio | RPLP2 | 1.0 |
|  | CTNNB1, DDR1, EIF4B | .9 |
|  | KDELR2, HDAC3, DDX5 | .8 |
|  | FIS1, EMP3, TNNC1, IFRD2, PDE6D, SEC61A1, ELOF1, DDX50, PRDX1 | .7 |
| Info Gain | RPLP2 | 1.0 |
|  | FIS1, EIF4B | .9 |
|  | KDELR2, HDAC3, DDX5, CTNNB1, DDR1 | .8 |
|  | SEC61A1, ELOF1 | .7 |
