# Supplementary material for: Cross-Species Meta-Analysis of Transcriptomic Data in Combination With Supervised Machine Learning Models Identifies the Common Gene Signature of Lactation Process
Source: Front Genet. 2018 Jul 12;9:235. doi: 10.3389/fgene.2018.00235 (PMC6052129; doi:10.3389/fgene.2018.00235)
Supplement: TABLE S2 — The results of application of 10 different attribute weighting algorithms for rat. [file Table_2.DOCX]

| **Model** | **Attribute** | **Weight** |
| --- | --- | --- |
| PCA | C1QB | 1.0 |
|  | CTSD | 0.8 |
|  | CTNNB1, RPS28 | 0.7 |
| SVM | CTSH, PNPLA6 | 1.0 |
|  | POMGNT1, LUM, STMN1, UHRF1, PDE6D, DSTN, ANXA11, IFRD2, THTPA | 0.9 |
|  | COL6A2, SPRY1, CLIC1, TKT, GSTP1, ATP5G3, ELOVL5, FTH1, CXCR4, AK1, FKBP4, MRPS18B | 0.8 |
|  | CTSD, FLNA, MYL2, IGJ, BRWD1, MESDC2 | 0.7 |
| Relief | RHEB | 1.0 |
|  | RPL27, RPL24, RPLP2, | 0.9 |
|  | ATP5G1, SLC25A11, NDUFV3, RPL18A | 0.8 |
|  | ELOF1 | 0.7 |
| Uncertainty | DDX56, SLC39A7, CTSA, FOLR2 | 1.0 |
|  | RPL27, SLC25A11, FTH1, GPI, RPL3, PDE6D, CALR, COL6A2, IGFBP2, NUDT2, PSMD6, ATP5G3, ADRBK1, CLDN3, LEO1, PGRMC1, OGN | 0.9 |
|  | RPS3, RPL10, VAMP8, AHCY, DSTN, HDAC3, RPL4, PSMB4, GNB2L1, LMNA, UQCRC2, PLP2, G6PD, C1QBP, GLTSCR2, ACAT2, EIF4A1, FKBP4, CTSH, CCT4, CLIC1, NFKBIA, ACTR1A, GPAA1, RSU1, TKT, ADD3, NUMA1, ADSL, AP3B1, SP1, YARS, POMGNT1, ALAS2, SPAG7, THTPA, FUS, MRPS18B, CTSD, TPP1, PDIA5, PLXNA2, C1D, LUM, CTNNB1, AKR1B1, C1QB, ELOVL5, STMN1, SPRY1, SCG5, IGFBP6, SF1, CD44 | 0.8 |
|  | GSTP1, EIF5A, PPP2R1A, RWDD1, DDX50, PRDX2, IER2, SMARCB1, SMARCB1, METAP2, IFRD2, GOT2, YY1, ID2, DDX5, RHOA, CCT5, CNOT7, USP12, SLC35C2, SFMBT1, LPL, NUDCD2, PSMC4, DERL1, NPC2, RPN2, BAT1, UHRF1, CTSC, ABHD10, DRG1, C3, CTSZ, MESDC2, ARHGAP29, HSPA8, AK1, SEC61A1, MYL2, SPNS1, RPS28, RTN4, ALPL, NUCB1, FGD1, SDAD1, PNPLA6 | 0.7 |
| Gini Index | DDX56, CTSA, FOLR2, RPL27, FTH1, GPI, RPL3, PDE6D, PSMD6, ATP5G3, ADRBK1, LEO1, PGRMC1, OGN, CALR, COL6A2, RPS3, VAMP8, DSTN, GNB2L1, UQCRC2, C1QBP, EIF4A1, FKBP4, CTSH, CCT4, CLIC1, TKT, ADSL, YARS, POMGNT1, ALAS2, SPAG7, THTPA, MRPS18B, CTSD, PLXNA2, LUM, CTNNB1, C1QB, ELOVL5, STMN1, SPRY1, SF1, EIF5A, RWDD1, IFRD2, CCT5, USP12, SFMBT1, PSMC4, UHRF1, CTSC, MESDC2, AK1, MYL2, RPS28, SDAD1, PNPLA6, EEF1B2, NDUFV2, MTCH1, RNH1, CCDC22, IGJ, IMMT, EEF1D, PPIB, SREBF1 | 1.0 |
| Chi Squared | DDX56, CTSA, FOLR2, RPL27, FTH1, GPI, RPL3, PDE6D, PSMD6, ATP5G3, ADRBK1, LEO1, PGRMC1, OGN, CALR, COL6A2, RPS3, VAMP8, DSTN, GNB2L1, UQCRC2, C1QBP, EIF4A1, FKBP4, CTSH, CCT4, CLIC1, TKT, ADSL, YARS, POMGNT1, ALAS2, SPAG7, THTPA, MRPS18B, CTSD, PLXNA2, LUM, CTNNB1, C1QB, ELOVL5, STMN1, SPRY1, SF1, EIF5A, RWDD1, IFRD2, CCT5, USP12, SFMBT1, PSMC4, UHRF1, CTSC, MESDC2, AK1, MYL2, RPS28, SDAD1, PNPLA6, SLC39A7, SLC25A11, NUDT2, CLDN3, IGFBP2, AHCY, HDAC3, RPL4, PSMB4, G6PD, NFKBIA, GPAA1, RSU1, ADD3, AP3B1, PDIA5, C1D, AKR1B1, IGFBP6, CD44, GSTP1, PPP2R1A, DDX50, METAP2, GOT2, YY1, ID2, CNOT7, SLC35C2, LPL, NUDCD2, DERL1, NPC2, BAT1, ABHD10, DRG1, CTSZ, ARHGAP29, HSPA8, NUCB1, FGD1, RPL10, LMNA, PLP2, GLTSCR2, ACAT2, ACTR1A, NUMA1, SP1, FUS, TPP1, SCG5, PRDX2, IER2, SMARCB1, DDX5, RHOA, RPN2, C3, SEC61A1, SPNS1, RTN4, ALPL | 1.0 |
| Deviation | CD44, SCG5, CXCR4 | 1.0 |
|  | HSPB1 | 0.9 |
|  | C1QB, IGFBP2 | 0.8 |
|  | C1S, CTSD, BRWD1 | 0.7 |
| Rule | CD44, SCG5, CXCR4, HSPB1, C1QB, IGFBP2, C1S, CTSD, STMN1, SPRY1, FLNA, ELOVL5, EIF4B, RPS28, CCT6A, CTNNB1, GCLC, NR2F1, TKT, C1D, NUCB1, FGD1, SDAD1, IGFBP6, COL6A2, PNPLA6, CTSA, CALR, C3, RAP1GDS1, EMP3, OGT, PDLIM7, MRM1, SF1, POMT1, NR4A1, NFIA, P4HA1, TAGLN2, CLDN3, MRPS18B, RTN4, RPS25, SPAG7, SPNS1, ALPL, RPN2, AKR1B1, POLE3, PDIA5, YARS, FOLR2, HES5, PGRMC1, PIM1, ZFP36, LEO1, SEC61A1, FOS, RBMS1, LPL, FUS, NFKBIA, NPC2, SREBF1, THTPA, TPP1, CTSZ, EIF4A1, KDELR2, AUP1, DRG1, OAT, ADRBK1, ATP5G3, EHF, CTSC, XDH, ARHGAP29, ADSL, DERL1, SP1, PSMC4, DPP3, SCARB1, CLU, GLT8D1, B4GALT2, LGTN, RHOA, BAT1, HGF, POMGNT1, ABT1, CLIC1, MESDC2, ADD3, PLA2G1B, C1QBP, FKBP4, ACAT2, NUMA1, CCT5, CCDC22, DDR1, IGJ, AP3B1, LAS1L, PPIB, CTSH, RNH1, SFMBT1, CCT4, G6PD, USP12, ID2, FIS1, TNNC1, SNW1, GLTSCR2, ANXA11, ACTR1A, CNOT7, PLD3, DDX56, SLC35C2, DDX5, RPL3, PRDX1, GAK, PLP2, UQCRC2, GNB2L1, PSMD6, MTCH1, FTH1, METAP2, LDHA, NUBP2, PRDX5, RSU1, LMNA, RPL4, PSEN2, IMMT, NUDT2, PRDX2, ATP2C1, SLC39A7, IFRD2, CSNK1A1, GPI, GPAA1, IER2, RBM9, PDE6D, GPX1, MOGAT1, EIF5A, TEX261, RPL7A, NEDD8, NDUFV2, LUM, RPL10, PPP2R1A, VPS25, GHITM, DSTN, EEF1B2, NAGK, VAMP8, RPS3, EEF1D, YY1, DAZAP2, SMARCB1, RWDD1, MYL2, HDAC3, PLXNA2, AK1, RPLP2, CAPZA2, OGN, AHCY, RPL27, NDUFV3, ATP5G1, ELOF1, DDX50, RPL24, SLC25A11, CORO1B, ALAS2, UHRF1, RHEB | 1.0 |
| Info Gain Ratio | C1QB, CTSD, STMN1, SPRY1, ELOVL5, RPS28, CTNNB1, TKT, SDAD1, COL6A2, PNPLA6, CTSA, CALR, SF1, MRPS18B, SPAG7, YARS, FOLR2, PGRMC1, LEO1, SREBF1, THTPA, EIF4A1, ADRBK1, ATP5G3, CTSC, ADSL, PSMC4, POMGNT1, CLIC1, MESDC2, C1QBP, FKBP4, CCT5, CCDC22, IGJ, PPIB, CTSH, RNH1, SFMBT1, CCT4, USP12, DDX56, RPL3, UQCRC2, GNB2L1, PSMD6, MTCH1, FTH1, IMMT, IFRD2, GPI, PDE6D, EIF5A, NDUFV2, LUM, DSTN, EEF1B2, VAMP8, RPS3, EEF1D, RWDD1, MYL2, PLXNA2, AK1, OGN, RPL27, ALAS2, UHRF1 | 1.0 |
| Info Gain | C1QB, CTSD, STMN1, SPRY1, ELOVL5, RPS28, CTNNB1, TKT, SDAD1, COL6A2, PNPLA6, CTSA, CALR, SF1, MRPS18B, SPAG7, YARS, FOLR2, PGRMC1, LEO1, SREBF1, THTPA, EIF4A1, ADRBK1, ATP5G3, CTSC, ADSL, ADSL, PSMC4, POMGNT1, CLIC1, MESDC2, C1QBP, FKBP4, CCT5, CCDC22, IGJ, PPIB, CTSH, RNH1, SFMBT1, CCT4, USP12, DDX56, RPL3, UQCRC2, GNB2L1, PSMD6, MTCH1, FTH1, IMMT, IFRD2, GPI, PDE6D, EIF5A, NDUFV2, LUM, DSTN, EEF1B2, VAMP8, RPS3, EEF1D, RWDD1, MYL2, PLXNA2, AK1, OGN, RPL27, ALAS2, UHRF1 | 1.0 |
