# Supplementary material for: Cross-Species Meta-Analysis of Transcriptomic Data in Combination With Supervised Machine Learning Models Identifies the Common Gene Signature of Lactation Process
Source: Front Genet. 2018 Jul 12;9:235. doi: 10.3389/fgene.2018.00235 (PMC6052129; doi:10.3389/fgene.2018.00235)
Supplement: TABLE S3 — The results of application of 10 different attribute weighting algorithms for Wallaby. [file Table_3.DOCX]

Table S3: The result of application of 10 different attribute weighting algorithms for Tammar Wallaby

| **Model** | **Attribute** | **Weight** |
| --- | --- | --- |
| PCA | G6PD | 1.0 |
|  | RPL18A, FTH1 | 0.9 |
| SVM | ADSL | 1.0 |
| Relief | PSPH, RBMS1 | 1.0 |
|  | FKBP4 | 0.9 |
|  | PSEN2, CTSA, THTPA, FUS | 0.8 |
|  | CXCR4, PLP2, ADRBK1 | 0.7 |
| Uncertainty | PRDX5 | 1.0 |
|  | PLXNA2, FOS, DPP3 | 0.9 |
|  | USP11, SLC35C2, RPL18A, FTH1, CLIC1, CCT5 | 0.8 |
|  | PLD3, AHCY, PDE6D, IGFBP6, FOLR2, ELOF1, RPS12 | 0.7 |
| Gini Index | GPAA1 | 1.0 |
| Chi Squared | PRDX5 | 1.0 |
|  | PLXNA2, CCT5, SLC35C2 | 0.7 |
| Deviation | AP3B1 | 1.0 |
|  | G6PD | 0.9 |
|  | RPL18A, FTH1 | 0.8 |
| Rule | IGFBP6, ADSL, AHCY, PSEN2, MTCH1, RBMS1 | 1.0 |
|  | GPAA1 | 0.8 |
| Info Gain Ratio | GPAA1 | 1.0 |
| Info Gain | GPAA1 | 1.0 |
|  | KDELR2 | 0.9 |
